# Temperature dependence of nitrogen solubility in bridgmanite and evolution of nitrogen storage capacity in the lower mantle

**DOI:** 10.1038/s41598-023-30556-5

**Published:** 2023-03-02

**Authors:** Ko Fukuyama, Hiroyuki Kagi, Toru Inoue, Sho Kakizawa, Toru Shinmei, Yuji Sano, Cécile Deligny, Evelyn Füri

**Affiliations:** 1 Geochemical Research Center, Graduate School of Science, The University of Tokyo, Hongo, Tokyo, 113-0033 Japan; 2 Geodynamics Research Center, Ehime University, Matsuyama, Ehime 790-5877 Japan; 3 Department of Earth and Planetary Systems Science, Hiroshima University, Higashi-Hiroshima, Hiroshima 739-8526 Japan; 4 Atmosphere and Ocean Research Institute, The University of Tokyo, Kashiwa, Chiba 277-8564 Japan; 5 CNRS, CRPG, Université de Lorraine, 54000 Nancy, France; 6 Present Address: Japan Synchrotron Radiation Research Institute, Hyogo, 679-5198 Japan; 7 Present Address: Center for Advanced Marine Core Research, Kochi University, Nankoku, Kochi 783-8502 Japan; 8 Present Address: Department of Geosciences, Swedish Museum of Natural History, Stockholm, Sweden

**Keywords:** Solid Earth sciences, Geochemistry, Mineralogy

## Abstract

Relative nitrogen abundance normalized by carbonaceous chondrites in the bulk silicate Earth appears to be depleted compared to other volatile elements. Especially, nitrogen behavior in the deep part of the Earth such as the lower mantle is not clearly understood. Here, we experimentally investigated the temperature dependence of nitrogen solubility in bridgmanite which occupies 75 wt.% of the lower mantle. The experimental temperature ranged from 1400 to 1700 °C at 28 GPa in the redox state corresponding to the shallow lower mantle. The maximum nitrogen solubility in bridgmanite (MgSiO_3_) increased from 1.8 ± 0.4 to 5.7 ± 0.8 ppm with increasing temperature from 1400 to 1700 °C. The nitrogen storage capacity of Mg-endmember bridgmanite under the current temperature conditions is 3.4 PAN (PAN: mass of present atmospheric nitrogen). Furthermore, the nitrogen solubility of bridgmanite increased with increasing temperature, in contrast to the nitrogen solubility of metallic iron. Thus, the nitrogen storage capacity of bridgmanite can be larger than that of metallic iron during the solidification of the magma ocean. Such a “hidden” nitrogen reservoir formed by bridgmanite in the lower mantle may have depleted the apparent nitrogen abundance ratio in the bulk silicate Earth.

## Introduction

Geochemical behavior of nitrogen in the deep Earth remains unclear, while many studies on the nitrogen cycle in the biosphere have been conducted to date^1–3^. Relative abundances of nitrogen, carbon, and H_2_O in BSE (bulk silicate Earth) normalized by those of carbonaceous chondrites are 0.11%, 1.49%, and 2.27%, respectively^4^. The BSE, which is assumed to be composed of the atmosphere, depleted mantle, and bulk mantle, is depleted in nitrogen compared to other volatile components^4^. The concentrations of ^14^N, ^12^C, and H_2_O in the carbonaceous chondrite composition used to estimate these abundance ratios were 1.09 × 10^–4^ mol/g, 2.94 × 10^–3^ mol/g, and 6.60 × 10^–3^ mol/g, respectively^5,6^. This apparently depleted nitrogen is referred to as “missing” nitrogen or “lost” nitrogen^4,7,8^. The nitrogen content in the BSE was estimated based on the Ar/N_2_ ratio. However, Zerkle and Mikhail^9^ argued that estimating the nitrogen abundance in BSE from the correlation between N_2_ and degassed Ar reported by Marty^10^ is not necessarily accurate. Marty^10^ assumed that nitrogen in the whole mantle exists as N_2_, which behaves like noble gas, but the oxygen fugacity in the deep mantle is much lower than the shallow mantle, and nitrogen exists as NH_3_, NH_4_^+^, or N^3−^ rather than N_2_^11,12^. High-pressure experiments showed that NH_4_^+^ can be incorporated into silicate minerals and silicate melts under reduced conditions^13–16^. Therefore, the “missing” nitrogen can also be explained by the existence of nitrogen reservoirs in the reduced deep mantle.

During the solidification of the magma ocean, degassing from the mantle magma ocean could be inefficient because the partially crystallized magma ocean behaved like a solid^17,18^. Therefore, the solidification of the magma ocean is suggested to be an important formation process of nitrogen reservoirs in the deep Earth^13,19^. Li et al.^13^ determined the nitrogen solubilities in forsterite and enstatite to be approximately 10 ppm and 100 ppm, respectively and suggested that the deep upper mantle can be a nitrogen reservoir through the solidification of the magma ocean. Yoshioka et al.^19^ experimentally determined nitrogen solubilities in wadsleyite and ringwoodite under high-pressure and high-temperature conditions corresponding to the mantle transition zone. The nitrogen solubilities in wadsleyite and ringwoodite ranged from 8.0 to 204.9 ppm and from 12.0 to 283.0 ppm, respectively, and these nitrogen solubilities increased with increasing temperature. Carbon solubilities in these mantle minerals were also investigated from high-pressure and high-temperature experiments followed by secondary ion mass spectrometry (SIMS) analysis^20^. Keppler et al.^21^ reported that carbon solubilities in olivine were up to 0.54 ppm and Shcheka et al.^20^ reported that those in wadsleyite and ringwoodite were below the SIMS detection limit (i.e., 30–200 ppb by weight). These mantle minerals, which have high N/C solubility ratios, can deplete nitrogen compared to carbon in BSE and can result in “missing” nitrogen. Yoshioka et al.^19^ conducted high-pressure experiments using multi-anvil apparatus under reduced conditions close to the Fe–FeO buffer and reported that nitrogen solubility in bridgmanite is 21.5 ± 18.1 ppm. However, their study determined the nitrogen solubility in bridgmanite only at a single condition of 24 GPa and 1600 °C, and the dependence of nitrogen solubility on temperature and chemical composition was not elucidated. The behavior of nitrogen, especially in the deep part of the Earth such as the lower mantle, remains unclear. Even in recent studies^22^, the deep segregation of volatiles caused by the solidification of magma ocean remains unconstrained. In this study, we conducted high-pressure and high-temperature experiments at different temperatures in the redox state corresponding to the lower mantle to investigate nitrogen incorporation into bridgmanite. Furthermore, the dependence of nitrogen solubility on the aluminum contents in bridgmanite was investigated in the same conditions.

## Results

### Descriptions of the run products

**Figure 1:**
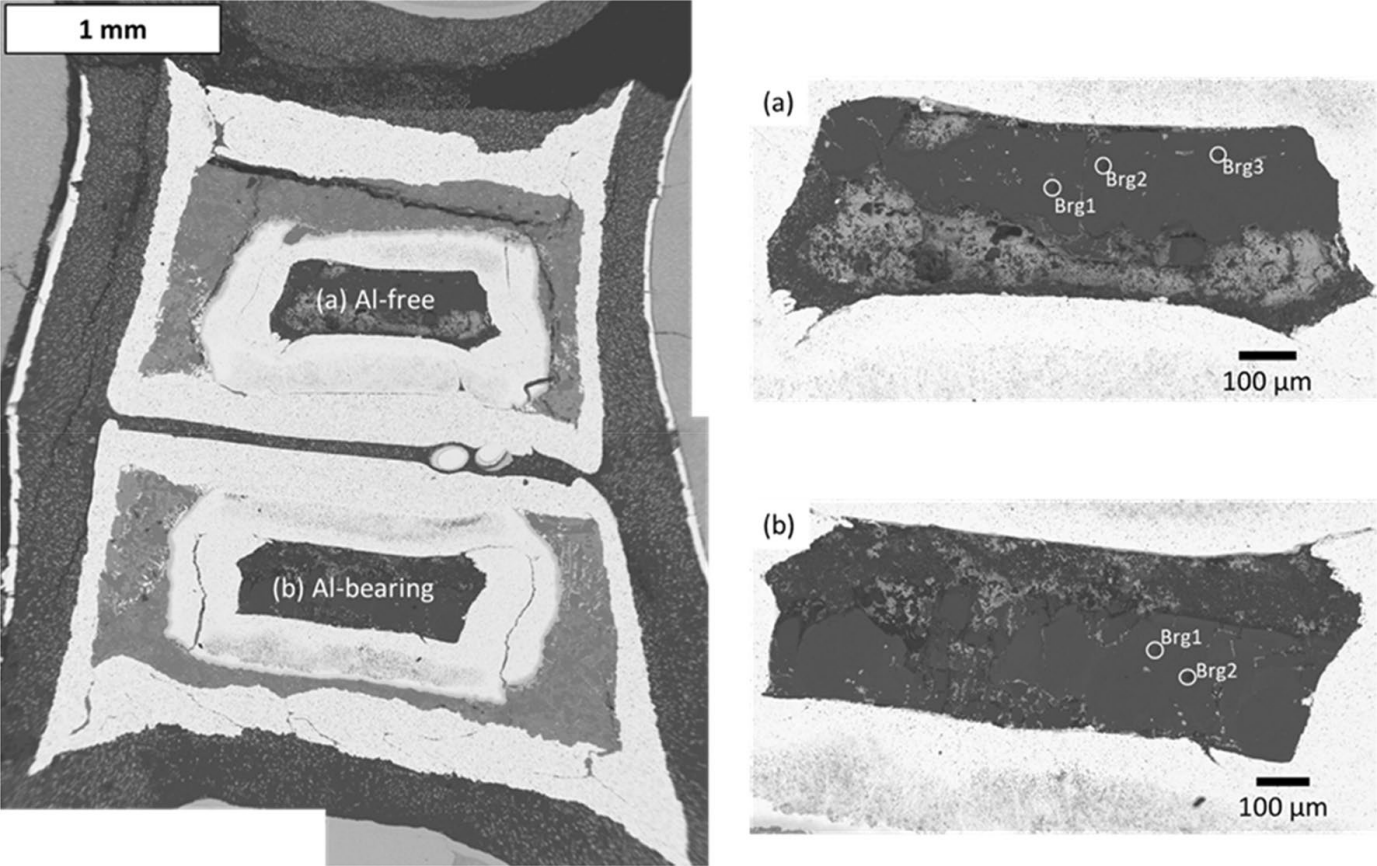
BSE images of recovered sample from 28 GPa, 1400 °C (OT2259). The left figure is a whole BSE image of the recovered sample. The right figures are the BSE images of two samples obtained by FE-SEM after high-resolution SIMS analysis; (**a**) Al-free system and (**b**) Al-bearing system. Circles correspond to analysis points. *Brg* bridgmanite.

Supplementary Table 2 lists the run products under experimental conditions of high-pressure and high-temperature experiments using the multi-anvil apparatus. All the run products contained crystals coexisting with hydrous melt (now present in the form of quenched crystals or glass) because ^15^N–substituted ammonium nitrate (^15^NH_4_^15^NO_3_) released water at high temperatures. The hydrous melts may have lost nitrogen during the quenching process and were not analyzed by SIMS. As reported by Yoshioka et al.^19^, quench crystals derived from hydrous melt coexisting with ^15^N–H–O fluid during cooling were observed in all the recovered samples. Roskosz et al.^23^ measured nitrogen solubility in peridotite melts under reduced conditions corresponding to the Fe–FeO buffer at high pressure and high temperature and found that nitrogen solubility in silicate melts increased with increasing pressure up to 3 GPa but plateaued from 3 to 14.8 GPa. This trend suggests that the nitrogen solubility in silicate melts above 3 GPa can be independent of pressure. Since the starting material in our study contains at least 60,000 ppm of nitrogen, which is approximately nine times the nitrogen solubility in the melt reported by Roskosz et al.^23^, all experiments in our study can be conducted under nitrogen-saturated conditions. As shown in Supplementary Fig. 3, the formation of bridgmanite was confirmed from previously reported Raman peaks^24^ in the recovered samples of the Al-free system, while some Raman spectra of the Al-bearing system showed broad bands indicating vitrification. Although some of the Al-bearing bridgmanite samples were vitrified during recovery from high pressure and high temperature, the experimental *P–T* conditions of the Al-bearing system were high enough to form bridgmanite, as demonstrated by other experimental results (see Supplementary Table 2). Therefore, it was assumed that the Al-bearing bridgmanite was stable during the experiments, but the possibility that nitrogen is released by the vitrification of Al-bearing bridgmanite cannot be ruled out. Figure 1 and Supplementary Figures 4–8 present the BSE images of the recovered cell assemblies including samples. In OT2258, OT2259, OT2293, OS3083, OT2474, and OT2515, no Fe–FeO leakage from the gold capsules was confirmed by SEM–EDS. In the Al-bearing systems of OT2258, OT2259, and OT2293, the recovered samples were contaminated with iron originating from the Fe–FeO buffer. These recovered samples affected by such iron contamination were not discussed in our research because the iron-contaminated samples could not reach equilibrium. After all these runs were completed, EDS analysis of the Fe–FeO buffers in the recovered samples revealed that pure metallic iron coexisted with FeO, which indicates that the oxygen fugacity conditions of the experiments were as low as those of the lower mantle. We inferred that hydrogen was generated in the outer gold capsule and permeated the inner platinum capsule. The MgO-rich hydrous melt in the inner platinum capsule is expected to contain ^15^NH_3_ in a hydrogen-coexisting environment. The obtained single crystals of bridgmanite were larger than 80 μm. These grain sizes were sufficiently large for SIMS analysis. Before SIMS analysis, we observed SEM–EDS images of the recovered samples, and the bridgmanite crystals and the grain boundaries were identified.

### Temperature dependence of nitrogen solubilities in bridgmanite (MgSiO_3_) and the chemical state of nitrogen

**Figure 2:**
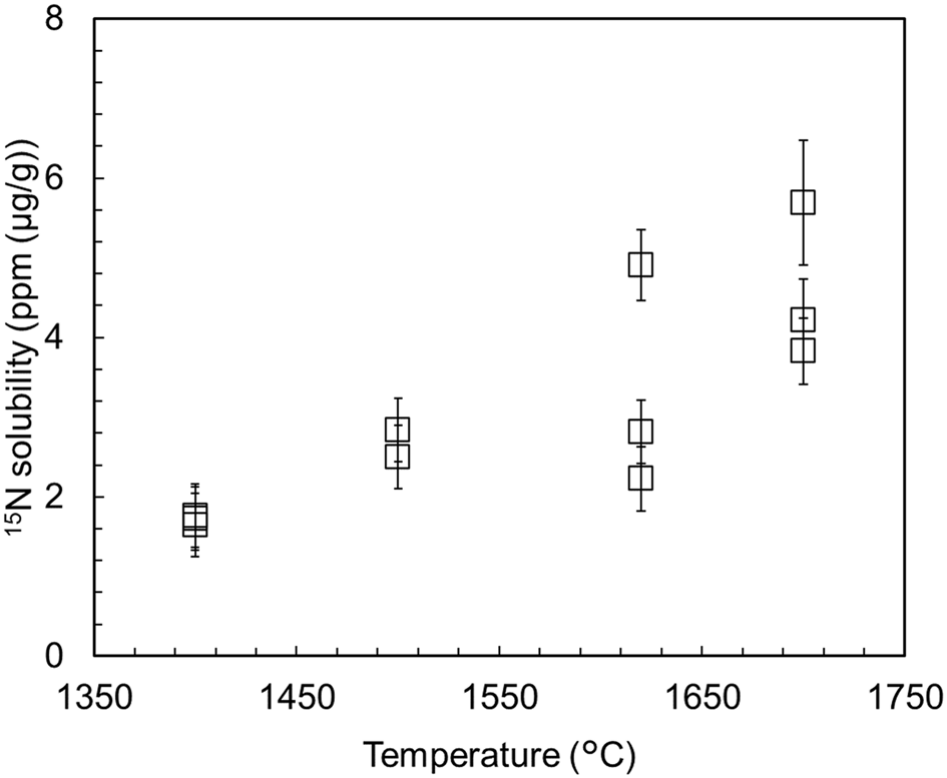
Plots depicting the nitrogen solubility in bridgmanite (MgSiO_3_) at different temperatures. All data points represent the single measurement points of the sample.

Figure 2 shows the nitrogen solubility in bridgmanite (MgSiO_3_) at various temperatures. Supplementary Table 3–8 lists chemical composition of run products and nitrogen concentrations of bridgmanite obtained from high-resolution SIMS (1280 HR2). The maximum nitrogen solubility in bridgmanite increased from 1.8 ± 0.4 to 5.7 ± 0.8 ppm (μg/g) with increasing temperature in the Al-free system. Li et al.^13^ proposed following three possibilities for nitrogen species incorporated into enstatite: N^3−^ into oxygen vacancies, N_2_ as Schottky defects, and substitution of ammonium ions (NH_4_^+^)_M2_ and (M^3+^)_M1_ with (Ca^2+^)_M2_ and (Mg^2+^)_M1_. Liu et al.^25^ reported that oxygen vacancies in bridgmanite increase with increasing temperature; thus, the observed temperature dependence of nitrogen solubility suggests that nitrogen can be incorporated into the lattice defects of bridgmanite. The substitution of N^3−^ for O^2−^ requires some charge compensation. Overall, the nitrogen solubility in bridgmanite obtained in this study was not as high as that in the previous study by Yoshioka et al.^19^, where nitrogen solubilities in bridgmanite ranged from 5.8 to 53.9 ppm. However, the nitrogen solubilities determined in this study are in good agreement with the minimum values reported by Yoshioka et al.^19^. The difference in nitrogen solubility in bridgmanite between our study and Yoshioka et al.^19^ could be caused by the coexistence of ringwoodite in Yoshioka et al.^19^. Ringwoodite has high nitrogen solubility up to 283 (± 3.8) ppm^19^, which can lead to the different results from those of our research. Yoshioka et al.^19^ reported the coexistence of ringwoodite in the bridgmanite phase because they conducted a high-pressure experiment at 24 GPa corresponding to the boundary of ringwoodite ↔ ferropericlase + bridgmanite^26^. Contrastingly, at 28 GPa, where our high-pressure experiments were conducted, ringwoodite could not coexist with bridgmanite and did not increase and scatter the nitrogen solubility in bridgmanite. Iron incorporation into bridgmanite also may cause the difference of nitrogen solubilities in bridgmanite between Yoshioka et al.^19^ and our research. Yoshioka et al.^19^ mixed a starting material and a nitrogen source (95% ^15^N-substituted NH_4_NO_3_) with metallic iron as an Fe–FeO buffer and enclosed the mixture in the same platinum capsule. Therefore, the chemical composition of bridgmanite reported by Yoshioka et al.^19^ was averaged as (Mg_0.97_, Fe_0.03_)SiO_3_ whose iron content was higher than that of Mg-pure bridgmanite (MgSiO_3_) in this study.

**Figure 3:**
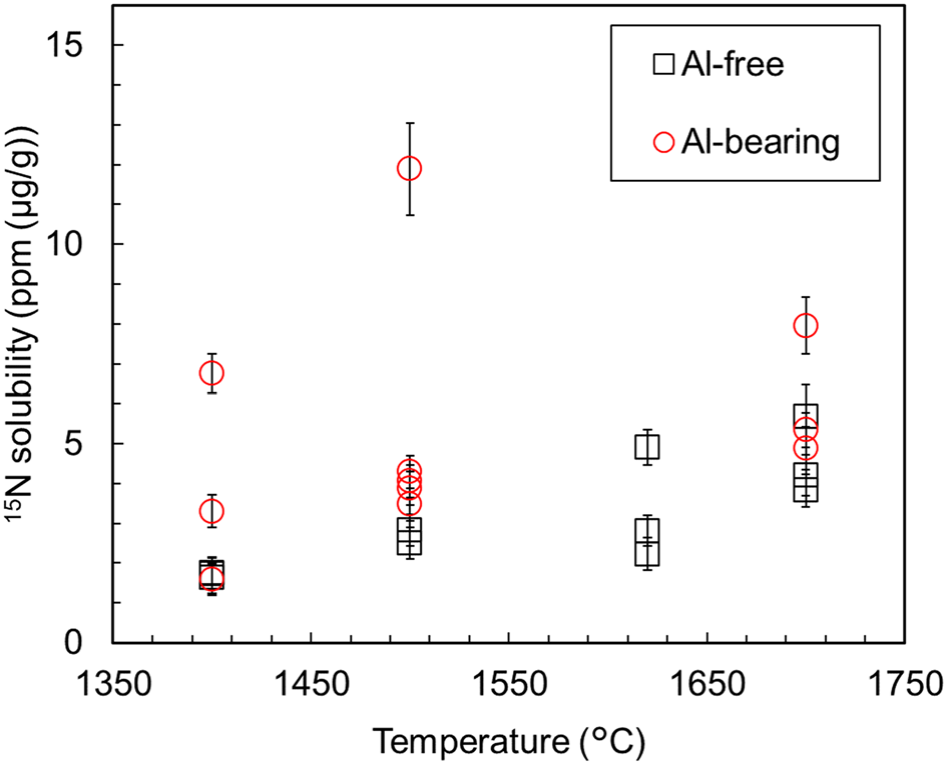
Plots showing the nitrogen solubility in bridgmanite at different temperatures. All data points represent the single measurement points of the sample.

**Figure 4:**
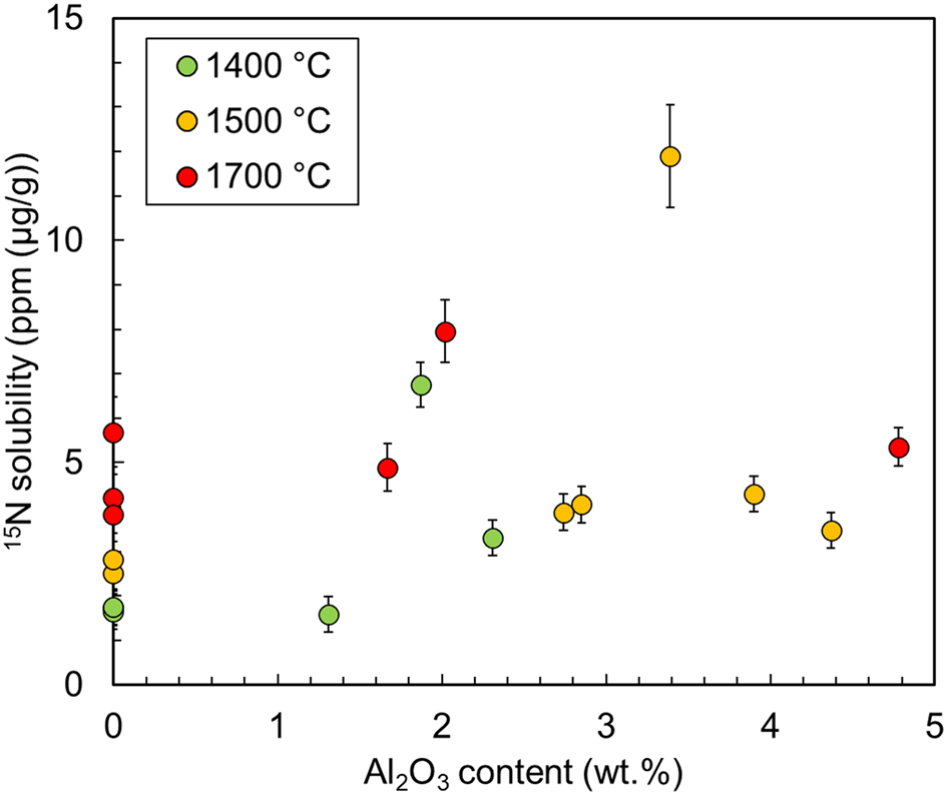
Plots of nitrogen solubility versus Al_2_O_3_ content in bridgmanite. All data points represent the single measurement points of the samples. The nitrogen solubilities in Al- and Fe-bearing bridgmanite are not shown here.

Figure 3 compares the solubility of nitrogen in Al-bearing bridgmanite and Al-free bridgmanite. Grüninger et al.^27^ reported that incorporation of aluminum into bridgmanite resulted in an increase in its oxygen vacancy by ~ 0.1 Al pfu (per formula unit). Figure 3 shows that the nitrogen solubility in Al-bearing bridgmanite is generally higher than that in Al-free bridgmanite. In our experiments, water was released from ^15^NH_4_^15^NO_3_ at high temperatures in all runs, and Si^4+^ ↔ Al^3+^ + H^+^ substitution in bridgmanite can occur^28^. Furthermore, we should consider a charge-coupled mechanism (as an AlAlO_3_ component). Therefore, the Al content in bridgmanite does not necessarily increase the oxygen defects and may not increase the nitrogen solubility. In fact, variations in Al_2_O_3_ content and nitrogen solubility in bridgmanite are not entirely systematic, as shown in Fig. 4. A partial substitution (N^3−^ + Si^4+^ ↔ O^2−^ + Al^3+^) can occur, but the systematic relationship between nitrogen solubility and Al_2_O_3_ content will be clarified if we can distinguish the three substitution mechanisms by theoretical simulations. This is an issue for the future, including how incorporated nitrogen exists in the crystal structure of bridgmanite.

## Discussion

We observed that the solubility of nitrogen in bridgmanite (MgSiO_3_) increased from 1.8 ± 0.4 to 5.7 ± 0.8 ppm with increasing temperature. Marty^4^ estimated the nitrogen concentration in the BSE to be 1.68 ppm based on the N/^40^Ar ratio of mid-ocean ridge basalts and plume-derived melts sampled at the Earth's surface, together with the presumed ^40^Ar (produced by the decay of ^40^K) content of the mantle. They pointed out the “missing” nitrogen, where nitrogen appears to be depleted by one order of magnitude compared to other volatile elements such as carbon and hydrogen when normalized to the carbonaceous chondrite composition. Shcheka et al.^20^ reported that the carbon solubility in bridgmanite was below the SIMS detection limit (i.e., below 30–200 ppb). These results suggest that bridgmanite (MgSiO_3_) has a high N/C solubility ratio (> 29) in considering 5.7 ppm nitrogen solubility of bridgmanite at 28 GPa and 1700 °C. The current nitrogen storage capacity in the Earth's present lower mantle is found to be 3.4 ± 0.5 PAN (PAN: Mass of present atmospheric nitrogen), given the same nitrogen solubility of 5.7 ± 0.8 ppm. If we attribute the cause of “missing” nitrogen solely to the lower mantle, the nitrogen storage capacity of the lower mantle should be at least 18 PAN (equivalent to 22.5 ppm nitrogen in the lower mantle). Consequently, the nitrogen storage capacity of the lower mantle estimated solely by the Mg-endmember bridgmanite (3.4 ± 0.5 PAN) cannot solve the “missing” nitrogen problem although this value is expected to be higher in the iron-bearing pyrolitic mantle. The nitrogen solubility in bridgmanite (MgSiO_3_) estimated in this study increased with increasing temperature. In this estimation, the pressure dependence is not considered and there can be a large uncertainty. The nitrogen solubility in bridgmanite is expected to increase with increasing pressure, similar to the nitrogen solubility of other mantle minerals such as olivine, wadsleyite, and ringwoodite^13,19^, but this is beyond the scope of our research. High-pressure experiments corresponding to the deeper part of the lower mantle are required to clarify pressure dependence in the future. Additionally, nitrogen solubility in ferropericlase, which occupies approximately 17 wt.% in the lower mantle^29^, should be investigated.

Although bridgmanite is the most abundant mineral in the lower mantle, other phases should be considered as potential nitrogen reservoirs in the Earth's interior. The solubility of nitrogen in metallic iron may be the highest in lower-mantle materials^19^. In fact, iron nitrides are discovered in super-deep diamonds derived from the lower mantle^7^.

**Figure 5:**
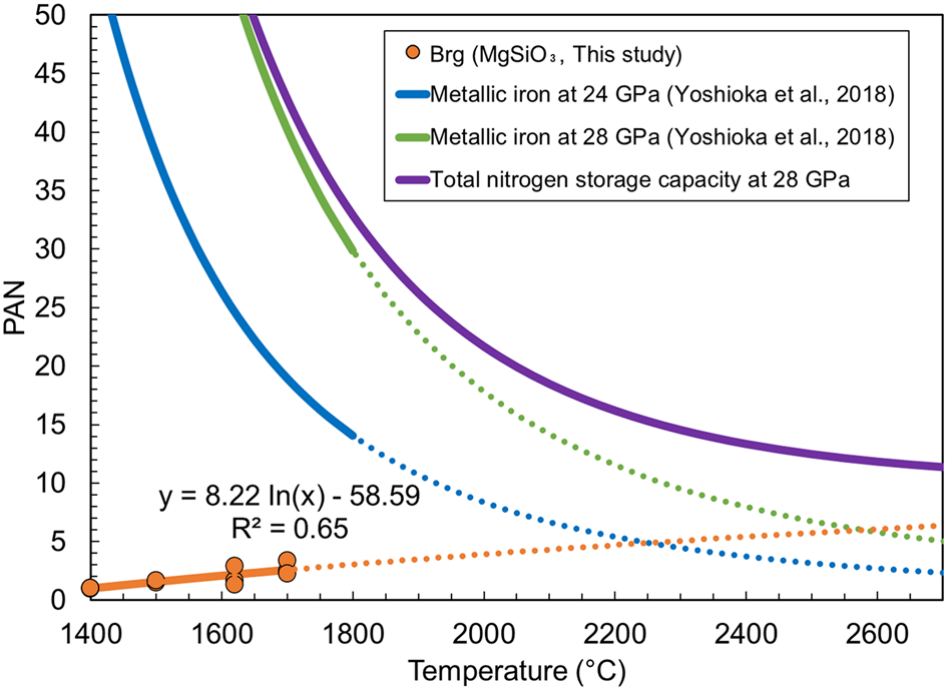
Plots exhibiting the temperature dependence of nitrogen storage in the lower mantle during cooling of the magma ocean. The temperature reaches 2700 °C, which is the melting point of bridgmanite at 28 GPa^40^. The mass of the present atmospheric nitrogen (PAN) is 3.92 × 10^18^ kg. In this estimation of nitrogen storage, the total mass of the Earth was assumed as 5.97 × 10^24^ kg, of which the lower mantle accounted for 52.2 wt.%.

In this study, the temperature dependence of nitrogen solubility in bridgmanite was demonstrated (at a constant pressure of 28 GPa), and the following logarithmic regression was applied using the least-squares method, as shown in Fig. 2. We obtained the following relation:

$$ c_{N} = 13.8 \cdot \ln T - 98.2, $$

where c_N_ is the nitrogen solubility in bridgmanite [ppm (µg/g)] and T is the temperature in Celsius. The coefficient of determination, R^2^, was 0.65. The experimental temperature range in this study was limited to 1400–1700 °C. Therefore, the fitted line was extrapolated to higher temperatures without considering the possible pressure dependence of nitrogen solubility. According to Liu et al.^25^, the number of oxygen vacancies of bridgmanite seems to increase linearly or logarithmically with increasing temperature, based on three experimental temperature points. In our discussion, we assume that oxygen vacancies increase logarithmically, but this assumption is not supported by theoretical evidence. An earlier study on oxygen vacancies in bridgmanite was limited to 2400 K^25^, and it is not clear whether our data can be applied up to 3000 K, but the nitrogen storage capacity of bridgmanite can reach 6.4 PAN at high temperature (2700 °C) such as those in magma oceans. On the other hand, nitrogen solubility of metallic iron as a function of temperature, pressure, and Fe content in Fe-Pt alloy was reported by Yoshioka et al.^19^ as follows:

$$\ln c_{N} = - 13.0 + 1.22 \cdot 10^{4} T^{ - 1} + 0.188P + 0.871x_{Fe},$$

where c_N_ is the nitrogen solubility of metallic iron (wt.%), T is the temperature in Kelvin, P is the pressure in GPa, and x_Fe_ is the molar fraction of Fe in the Fe–Pt alloy. The coefficient of determination, R^2^, is 0.82. Yoshioka et al.^19^ reported that the solubility of nitrogen in metallic iron decreased with increasing Pt content. Pt was derived from the sample capsule. Natural metallic iron in the lower mantle may contain Pt, but its concentration should be very low. In our study, we assumed that there was no Pt in metallic iron in the lower mantle. In the absence of Pt, the solubility of N in metallic Fe increases. Since the experimental pressure and temperature ranges for this regression fit are from 14 to 24 GPa and 1100 to 1800 °C, respectively^19^, the nitrogen solubility in metallic iron needs to be extrapolated to higher temperatures and pressures to assess the nitrogen storage capacity of the whole terrestrial lower mantle. Yoshioka et al.^19^ estimated nitrogen solubility in metallic iron in the whole lower mantle by applying a regression fit at 24 GPa; the same estimation method was used in this study. From the regression fits of the nitrogen solubility in bridgmanite determined in this study and the nitrogen solubility in metallic iron determined by Yoshioka et al.^19^, we obtained the nitrogen storage capacity in Earth's lower mantle as a function of temperature (see Fig. 5). Here, the metallic iron and bridgmanite contents in the lower mantle estimated from the pyrolite model were assumed to be 1 wt.% and 75 wt.%, respectively, as reported in the previous studies^29,30^. The nitrogen storage capacity of metallic iron is larger than that of bridgmanite at low temperatures, such as in the current mantle; however, the relationship of nitrogen solubility between metallic iron and bridgmanite is reversed at approximately 2200 °C (see Fig. 5). Yoshioka et al.^19^ reported a pressure dependence of nitrogen solubility in metallic iron; when considering the nitrogen solubilities in bridgmanite and metallic iron at 28 GPa, the nitrogen solubility relationship between metallic iron and bridgmanite is reversed at around 2600 °C.

Roskosz et al.^23^ reported the nitrogen solubility in peridotite melt under high pressures (1.8‒17.7 GPa) and high temperatures (2350‒2800 K) and the nitrogen solubility plateaued at approximately 7 × 10^3^ ppm under pressures above 5.0 GPa. To estimate the nitrogen concentration in bridgmanite through solidification of the magma ocean, we need to determine the partition coefficient between bridgmanite and magma. In the present study, nitrogen concentration in the melt was not determined and a pseudo-partition coefficient was obtained from the ratio of nitrogen solubilities of bridgmanite to that of peridotite melts. The nitrogen solubility of bridgmanite obtained in this study was 5.7 ppm, which is its maximum solubility at 1700 °C. Nitrogen solubility in the peridotite melt was considered to be 7 × 10^3^ ppm by extrapolating the results from Roskosz et al.^23^ to 28 GPa. By dividing the nitrogen solubility in bridgmanite by that of the peridotite melt, the D_bridgmanite/silicate melt_ was estimated to be 8 × 10^–4^. In the estimated pseudo-partition coefficient, we did not consider structure changes of melt with increasing pressure^31^ and pressure dependence of nitrogen solubility in mantle minerals^13,19^. Therefore, there are unmeasurable uncertainties in the coefficient. Assuming that the nitrogen content of the magma ocean is that of CI chondrites (1235 ± 440 μg/g)^32^, the mass of nitrogen retained only by bridgmanite in the lower mantle was 0.6 PAN. Although enstatite chondrites have recently been favored for early Earth materials^33^, we assumed a CI chondrite for early Earth composition based on the estimation by Marty^4^. This mass of nitrogen in the lower mantle is too small to evaluate the “missing” nitrogen because the lower mantle needs to store more than 18 PAN as mentioned before. In this estimation, we did not consider the pressure dependence of nitrogen solubility in lower-mantle materials. Furthermore, this estimation does not consider the loss of nitrogen to the atmosphere, and the mass of retained nitrogen can be lower than 0.6 PAN. The obtained nitrogen storage capacity in the lower mantle can be underestimated because the nitrogen solubility in mantle minerals such as olivine, wadsleyite, and ringwoodite increases with increasing experimental pressure^13,19^, as described previously. It was assumed that the mass ratios of bridgmanite and metallic iron were constant. The temperature dependence of the nitrogen solubility in Ca-perovskite and ferropericlase is expected to be determined in the future.

The ability of bridgmanite to capture nitrogen depends on the scenario of magma ocean convection. Before discussing the nitrogen storage capacity, we need to consider following two cases: (1) full convection of the magma ocean; and (2) crystallization of the magma ocean and cessation of convection. Assuming scenario (1), nitrogen in the magma ocean would be released into the primordial atmosphere and could hardly be retained. In this case, bridgmanite did not play a role in preserving nitrogen in the lower mantle. In contrast, while assuming scenario (2), nitrogen in the magma ocean can be retained in mineral phases. Miyazaki and Korenaga^34^ suggested that efficient degassing of the mantle is unlikely during the solidification of a magma ocean if a rheological transition in a partially molten medium is considered. In fact, Abe^17^ reported that hard magma ocean, which contains substantial amounts of melt (up to 30–40%), had solid-like viscosity. Solomatov^18^ also reported that the magma ocean behaved like a solid. Yoshioka et al.^19^ also suggested that the magma ocean essentially behaved as a closed system because such a magma ocean is too viscous for rapid convection above a threshold of 40–80% crystallization of the magma^35,36^. Furthermore, Xie et al.^37^ reported that an enriched bridgmanite layer can be formed first at the top of the lower mantle during crystallization of the magma ocean, which may have resisted mantle mixing by convection. In fact, Caracas et al.^38^ reported that the basal magma ocean, which is formed by crystallized bridgmanite that would float in the magma ocean, could be enriched in incompatible elements through the solidification of the magma ocean. These reports support the idea that the lower mantle could behave as a closed system through solidification of the magma ocean, and nitrogen in the magma ocean could be incorporated into bridgmanite up to its solubility. However, if we assume that over 99.2% of nitrogen was lost from the magma ocean, whose chemical composition is CI chondritic before the magma ocean becomes highly viscous, bridgmanite cannot capture nitrogen up to its solubility at high temperatures (~ 6.4 PAN). The temperature of the terrestrial magma ocean is estimated to be 3000–3500 K^39^, which is considerably higher than our experimental temperature conditions (1400–1700 °C). The nitrogen solubility in bridgmanite was extrapolated to approximately 2700 °C, which corresponds to the melting point of bridgmanite at 28 GPa^40^ and the lowest temperature of the magma ocean reported by Bouhifd and Jephcoat^39^. The nitrogen solubility in bridgmanite was extrapolated to higher temperatures by assuming that it increased logarithmically. As shown in Fig. 5, bridgmanite can be a significant nitrogen reservoir at higher temperatures such as solidification of the magma ocean, whereas metallic iron can be a significant nitrogen reservoir to solve “missing” nitrogen (18 PAN ~) at the present lower temperatures.

**Figure 6:**
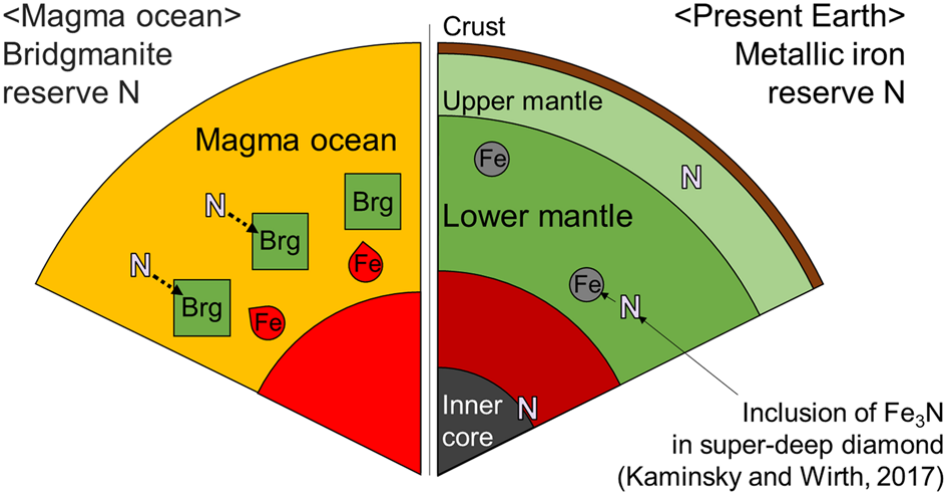
Schematic depicting the nitrogen reservoir formation processes in the lower mantle through solidification of the terrestrial magma ocean.

Overall, we found that bridgmanite plays an essential role in preserving nitrogen during the solidification of Earth's magma ocean. Metallic iron also plays a significant role in the retention of nitrogen in the lower mantle (Fig. 6). Although iron nitrides were discovered as inclusions of super-deep diamonds originating from the lower mantle^7^, Speelmanns et al.^41^ argued that these nitrides originated from the upper mantle because the nitrogen partition coefficients between the metal and silicate melts ($D_{N}^{metal\, melt/silicate\, melt}$) are lower than 1 under reduced conditions. However, nitrogen stored in bridgmanite can diffuse into metallic iron during mantle cooling because the solubility of nitrogen in metallic iron is much higher than that in bridgmanite^19^.

In this study, we experimentally determined nitrogen solubilities in bridgmanite under reduced conditions corresponding to the shallow lower mantle. Nitrogen solubilities in bridgmanite (MgSiO_3_) increased with the increase in temperature. The maximum nitrogen solubilities at 1400 °C, 1500 °C, 1620 °C, and 1700 °C were 1.8 ± 0.4 ppm, 2.8 ± 0.4 ppm, 4.9 ± 0.4 ppm 5.7 ± 0.8 ppm, respectively. Moreover, we observed that bridgmanite can play a major role in storing nitrogen in the hotter early lower mantle, whereas metallic iron can play a major role in storing nitrogen in the current colder lower mantle. Nitrogen solubility in bridgmanite did not show a clear increase with increasing Al_2_O_3_ content. The obtained results suggest that bridgmanite stores nitrogen in the lower mantle through the solidification of the magma ocean and metallic iron can inherit the nitrogen retained by bridgmanite in the lower mantle. To evaluate the nitrogen storage capacity of the lower mantle more accurately, we need to conduct experiments at higher pressures and determine nitrogen solubility in ferropericlase which is the second most abundant in the lower mantle in the future.

## Methods

### Starting materials

Two types of starting materials were prepared: powdered mixtures of (a) MgO and SiO_2_ (quartz) corresponding to the ideal bridgmanite composition; (b) Al_2_O_3_, MgO, Mg(OH)_2_, and SiO_2_ for an Al-bearing bridgmanite composition (see Supplementary Table 1). ^15^N–substituted ammonium nitrate (^15^NH_4_^15^NO_3_, isotopic purity > 99.6%, SHOKO SCIENCE Corp.) was used as a nitrogen source to distinguish nitrogen contained in the mineral samples from the nitrogen contamination induced by the experimental procedures. Nitrogen contamination is thought to originate from atmospheric nitrogen during sampling or from the resin used to mount the sample. Contamination by atmospheric ^15^N is negligible because the natural abundance of ^15^N is more than two orders of magnitude lower than that of atmospheric ^14^N (^15^N/^14^N = 3.65 × 10^–3^). The starting materials and ^15^NH_4_^15^NO_3_ were enclosed in a platinum capsule. The mass ratio of the starting materials to the nitrogen source was approximately 5:1 for each experiment (the molar ratio of the starting materials to the nitrogen source was also approximately 5:1). The starting material was separated from ^15^NH_4_^15^NO_3_ using gold foil with a thickness of 30 μm in Run No. OS3083. In the other runs, the starting material and ^15^NH_4_^15^NO_3_ were mixed.

### High-pressure and high-temperature experiments

**Figure 7:**
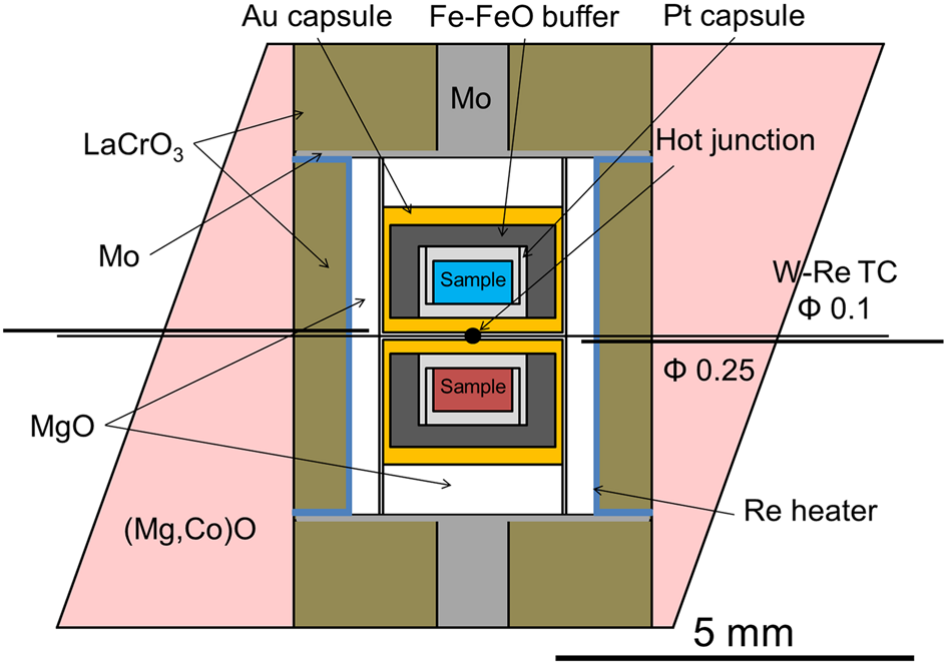
Schematic illustrating the cell assembly that was used in high-pressure and high-temperature experiments using multi-anvil apparatus. A LaCrO_3_ (brown) sleeve served as a thermal insulator. A platinum (light gray) sample capsule was made by combining two platinum tubes with 0.1 mm wall thickness, and outer diameters of 1.3 mm and 1.5 mm, respectively, by welding each end of the capsules. A gold capsule (yellow) was made from a gold tube with 0.1 mm wall thickness and 2.5 mm outer diameter.

High-pressure and high-temperature experiments were conducted using Kawai-type 2000-ton multi-anvil apparatus (Orange-2000) and Kawai-type 3000-ton multi-anvil apparatus (Orange-3000) installed at Geodynamics Research Center, Ehime University (GRC), Japan. The Orange-2000 was used only for Run No. OS3083, whereas all other experiments were conducted using the Orange-3000. All experiments were conducted at 28 GPa and temperatures were 1400 °C, 1500 °C, 1620 °C and 1700 °C, respectively (see also Supplementary Table 2). The relationship between the pressure and load was calibrated in advance. The heating duration for all experiments was 2 h. Tungsten carbide anvils (Fujilloy F08) with 4 mm truncated edge length (TEL) were used. The cell assembly used in this study is shown in Fig. 7. A platinum sample capsule was surrounded by an Fe-FeO buffer (iron wüstite buffer) to reproduce the oxygen fugacity corresponding to the lower mantle condition^30,42,43^. We used 150 mesh iron powder and iron oxide (FeO) powders with 8 μm or 200 mesh for the Fe-FeO buffer [Fe:FeO = 2:1 (wt. %)]. Then, 20–50 μl of water was added to 0.5 g of Fe–FeO buffer. The platinum capsule was enclosed in an outer gold capsule. The two gold capsules were insulated from the Re heater with a thickness of 25 μm using a magnesia sleeve. The temperature was measured with a precision of ± 5 °C using a W–Re (W3%Re–W25%Re) thermocouple inserted into the octahedron and attached to the gold capsules. The hydrogen fugacity in the inner and outer capsules was assumed to be equal because of the high hydrogen permeability of platinum compared with that of gold. ^15^NH_4_^15^NO_3_ decomposes into ^15^N_2_O and H_2_O at high temperatures, and ^15^NH_3_ is expected to be formed in the ^15^N–H–O fluid under reduced conditions buffered by Fe–FeO in an inner platinum capsule.

### Chemical analysis and phase determination (FE–SEM–EDS, FE-EPMA, and Raman spectroscopy)

Backscattered electron (BSE) images of the recovered samples were obtained using FE-SEM (JSM-7000F; JEOL) installed at Department of Earth and Planetary Science, The University of Tokyo. Chemical compositions of the quenched minerals were analyzed using the SEM–EDS (JSM-7000F; JEOL) under 15 kV and 87.4–130.4 μA before and after SIMS analysis. The deviation of the Mg/Si ratios of bridgmanite obtained from SEM–EDS analysis was corrected by FE-EPMA (JXA-8530F; JEOL) installed at Department of Earth and Planetary Science, The University of Tokyo. Six species of standards were used for the FE-EPMA analysis: garnet, enstatite, augite, plagioclase, San Carlos olivine, titanaugite olivine basalt, and JB-1 (silicate rock standards of the Geological Survey of Japan^44^).

To determine the mineral phases, Raman spectra were obtained using a micro-Raman spectrometer installed at Geochemical Research Center, The University of Tokyo, using an Ar ion laser with a wavelength of 514.5 nm and a power of 6 mW for excitation. The beam size was approximately 2 μm in diameter and the exposure time was 30 s.

### Secondary ion mass spectrometry (SIMS)

Quantitative analysis of the nitrogen in bridgmanite was conducted using high-resolution SIMS (1280 HR2, CAMECA) installed at Centre de Recherches Pétrographiques et Géochimiques, Université de Lorraine. The recovered samples were polished; those in the Al-free and Al-bearing systems were gold-coated. The primary ion beam was a 10 keV Cs^+^ beam with a current of ~ 10 nA and a spot size of ~ 40 μm. A normal-incidence electron gun was used for charge compensation during the analysis. The mass resolution (m/Δm) was ≈ 13,000, and the raster size was 5 μm × 5 μm. Samples were pre-sputtered for 180 s in 10 μm × 10 μm rasters to minimize surface contamination. Spot analyses of negatively charged ions were conducted at different mass stations (masses of 30, 31, and 32). For the nitrogen abundance measurements, the ^15^N^16^O^−^ molecular ion was analyzed for 25 cycles using an axial electron multiplier. The count times for detecting ^27^Al^−^, ^30^Si^−^, ^14^N^16^O^−^, ^15^N^16^O^−^, and ^16^O_2_^−^ by peak jumping were 4 s, 4 s, 6 s, 20 s, and 4 s, respectively, and the total analysis time was approximately 30 min. Nitrogen concentrations were obtained from the secondary ion intensity ratio ^15^N^16^O^–^/^16^O_2_^–^ for bridgmanite using a calibration line obtained from eight synthetic basaltic glasses with known nitrogen content^45^ (see Supplementary Fig. 2). Matrix-matched and homogeneous standards with variable N contents are not available in any SIMS laboratories; however, this method has been demonstrated to yield nitrogen (^14^N) abundances in silicate glasses with variable compositions (i.e., NBO/T), which are in excellent agreement with those obtained using static mass spectrometry, even for low N contents (≤ 1 ppm)^46^. Therefore, for glasses, any matrix effects on the yields of secondary molecular ions (NO^−^ and O^2−^) are considered negligible. Given the high mass resolution, we succeeded in separating ^15^N^16^O^−^ and ^29^SiH_2_^−^ ions, as shown in Supplementary Fig. 1, and directly estimated the N content based on the ion counts of ^15^N^16^O^−^ ions. In this study, bridgmanite with the lowest nitrogen solubility of 1.6 ± 0.4 ppm yielded an average ^15^N^16^O^−^ ion count rate of 8.3 cps. This count rate was much higher than that of the EM background (< 1 cps). Atmospheric nitrogen can contaminate starting materials during encapsulation. After the experiment, nitrogen originating from the resin used for the sample mounts can also contaminate the recovered samples. Nitrogen was the main component of the resin used in our study. In the nitrogen analysis, the targeted particles (> 80 µm) were considerably larger than the beam size (20 µm), avoiding grain boundaries. The ^14^N^16^O^−^ signal was also monitored, and it was inferred that the concentration of ^15^N originating from atmospheric contamination was smaller than the analytical errors and negligible, considering the natural nitrogen isotopic ratio (^15^N:^14^N = 0.0036:0.9964).

## Supplementary Information

Supplementary Information.

## Data Availability

The datasets used and/or analyzed during the current study are available from the corresponding author(s) on reasonable request.
