# Supplementary material for: Temperature dependence of nitrogen solubility in bridgmanite and evolution of nitrogen storage capacity in the lower mantle
Source: Sci Rep. 2023 Mar 2;13:3537. doi: 10.1038/s41598-023-30556-5 (PMC9981615; doi:10.1038/s41598-023-30556-5)
Supplement: Supplementary file 1 — Supplementary Information. [file 41598_2023_30556_MOESM1_ESM.pdf]

Supplementary information

Temperature dependence of nitrogen solubility in bridgmanite and evolution of nitrogen storage capacity in the lower mantle

Ko Fukuyama*[1, 2], Hiroyuki Kagi*[1], Toru Inoue[3], Sho Kakizawa[3,4], Toru Shinmei[2], Yuji Sano[5,6], Cécile Deligny[7,8], Evelyn Füri[7]

[1]Geochemical Research Center, Graduate School of Science, The University of Tokyo, Hongo, Tokyo 113-0033, Japan

[2]Geodynamics Research Center, Ehime University, Matsuyama, Ehime 790-5877, Japan

[3]Department of Earth and Planetary Systems Science, Hiroshima University, Higashi-Hiroshima, Hiroshima 739-8526, Japan

[4] Present address: Japan Synchrotron Radiation Research Institute, Hyogo, 679-5198, Japan

[5]Atmosphere and Ocean Research Institute, The University of Tokyo, Kashiwa, Chiba, 277-8564, Japan

[6]Present address: Center for Advanced Marine Core Research, Kochi University, Nankoku, Kochi, 783-8502, Japan

[7]Université de Lorraine, CNRS, CRPG, F-54000 Nancy, France

[8]Present address: Department of Geosciences, Swedish Museum of Natural History, Stockholm, Sweden

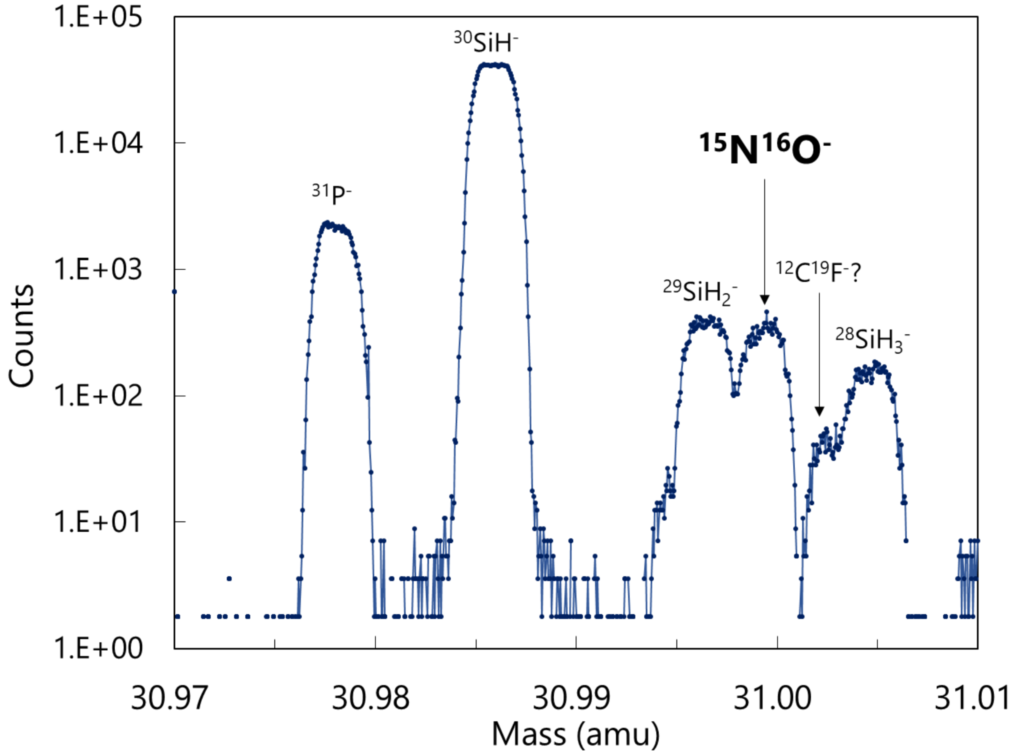

Supplementary Figure 1. High-resolution mass spectrum at mass station 31 obtained by SIMS (1280 HR2).

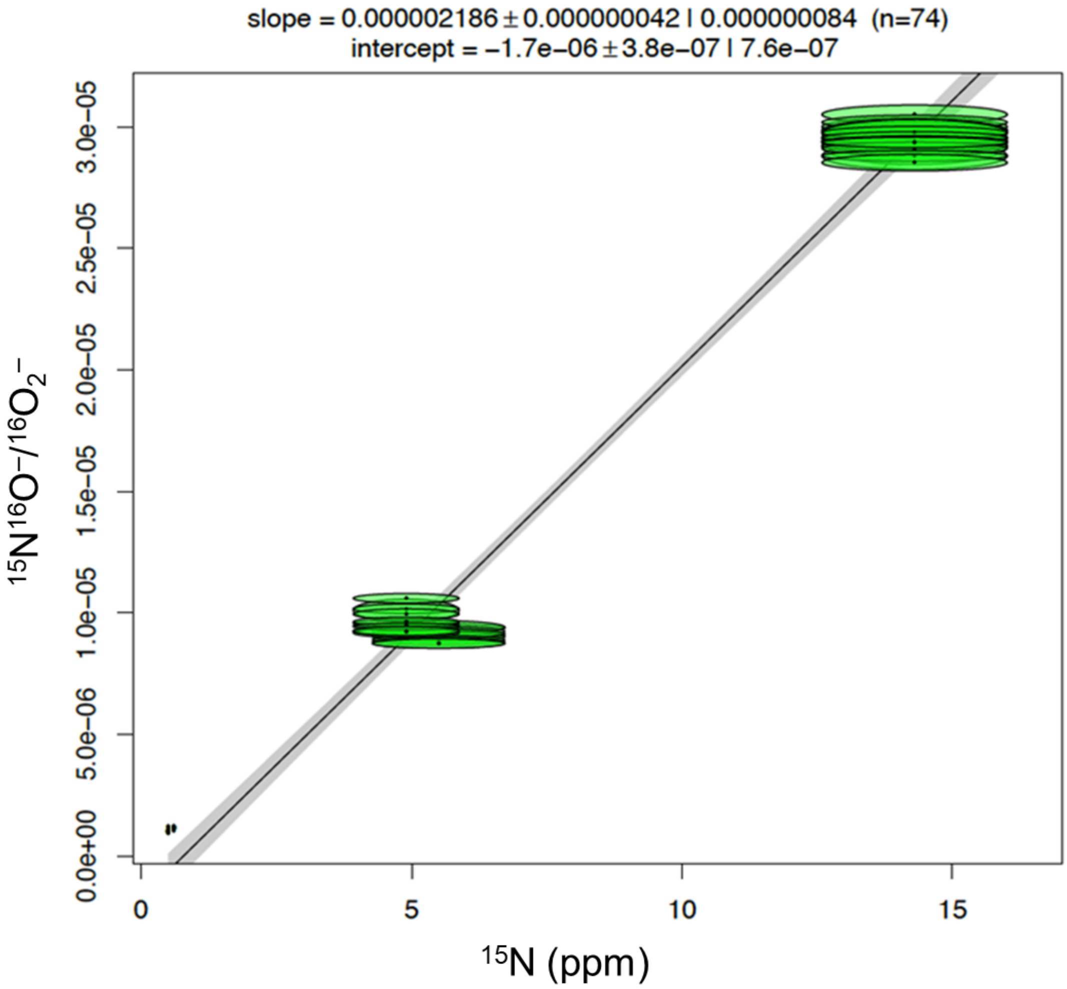

Supplementary Figure 2. Calibration line for estimating nitrogen concentrations in bridgmanite. This line was established using CM-1#4A, CM-1#1H, and CB-2#1 from Fűri et al. (2018) as standards.

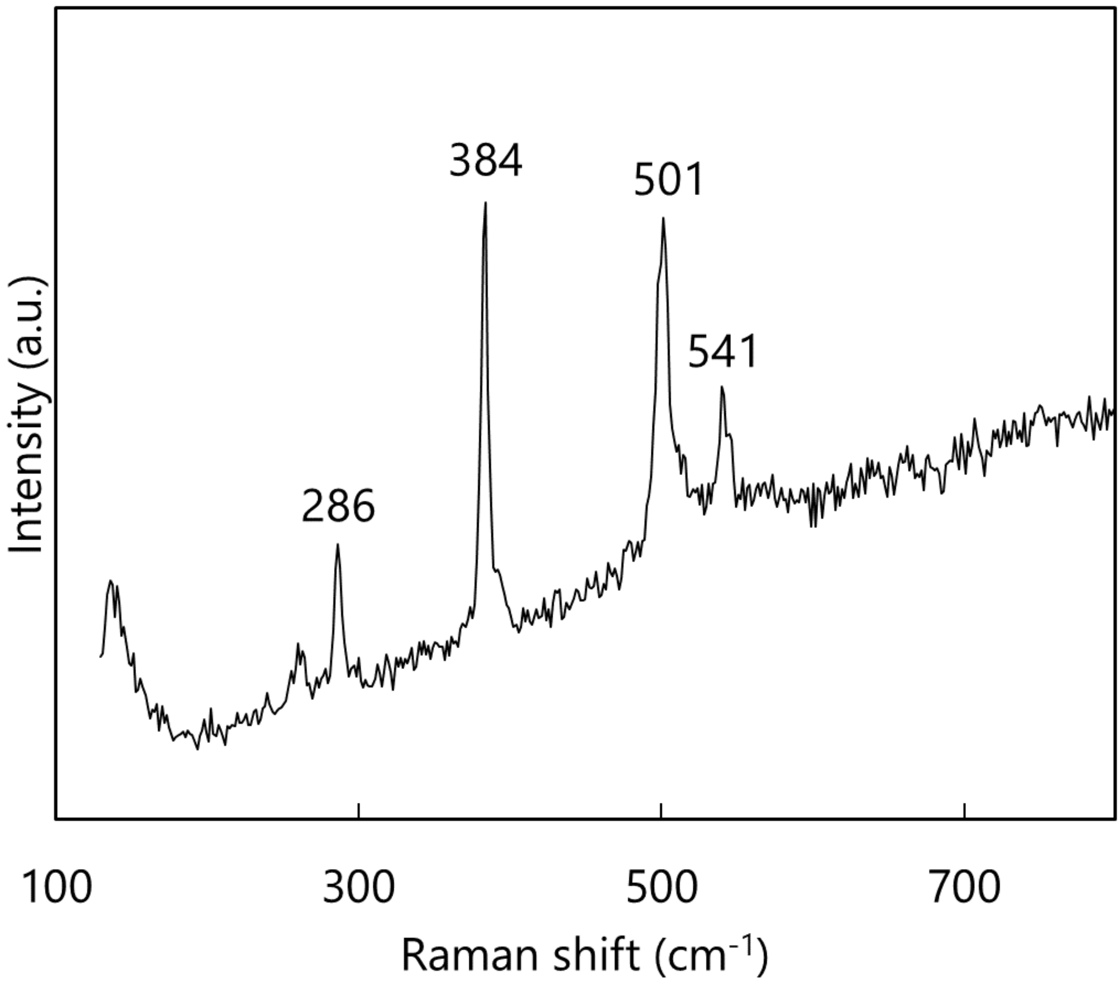

Supplementary Figure 3. Raman spectrum of a recovered Al-free bridgmanite (OS3083). Raman peaks of 282 cm^{-1}, 382cm^{-1}, 501 cm^{-1}, and 541 cm^{-1} are representative as reported in Gillet et al. (2000).

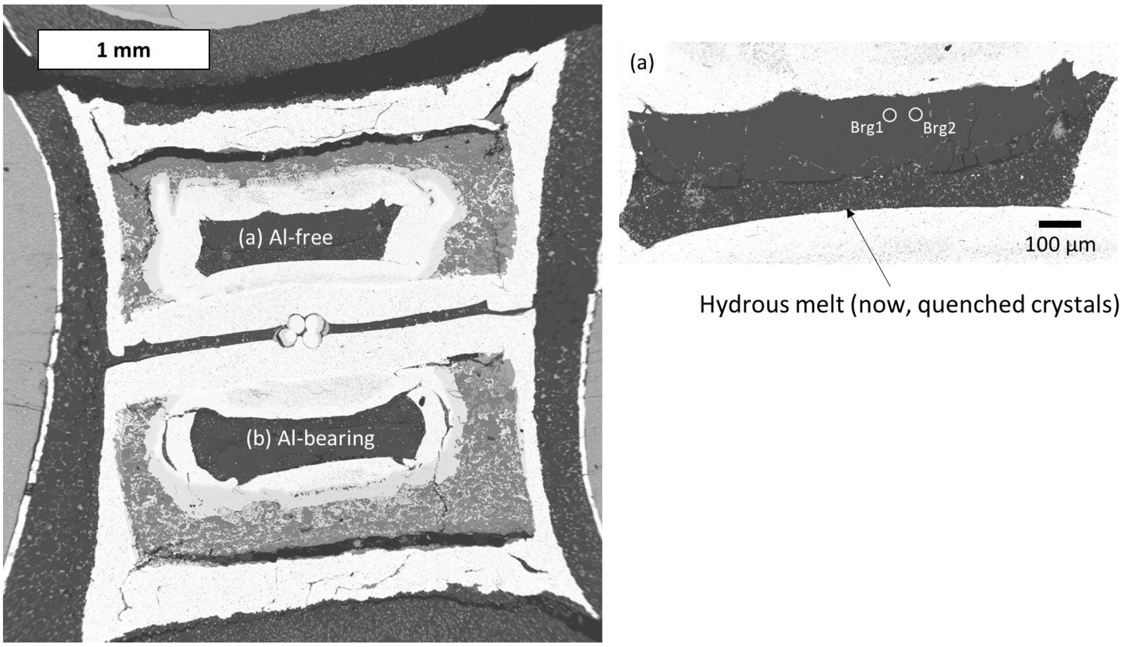

Supplementary Figure 4. Backscattered electron (BSE) images of sample OT2258 synthesized at 28 GPa and 1500 °C. Left: BSE image of the entire recovered sample. Right: BSE image of the Al-free sample obtained by FE-SEM after high-resolution SIMS analysis. Circles correspond to the analysis points. Brg, bridgmanite.

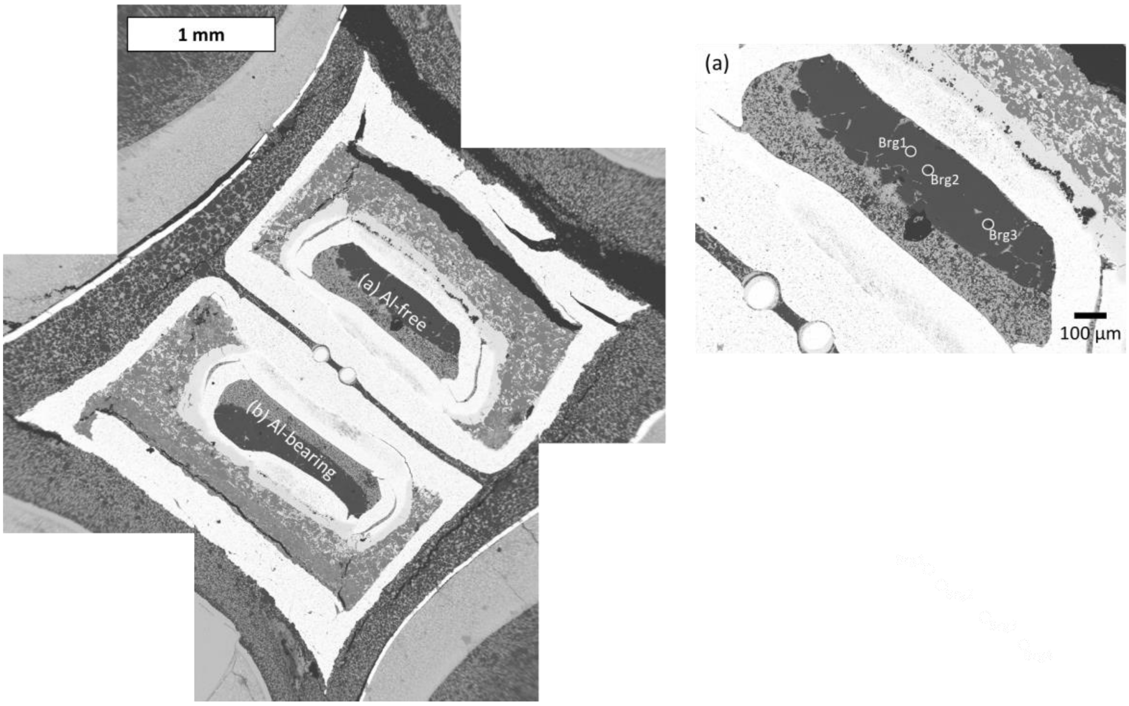

Supplementary Figure 5. BSE images of recovered sample from 28 GPa, 1620 °C (OT2293). The left figure is a whole BSE image of the recovered sample. The right figure is the BSE images of Al-free samples obtained by FE-SEM after high-resolution SIMS analysi. Circles correspond to analysis points. Brg, bridgmanite.

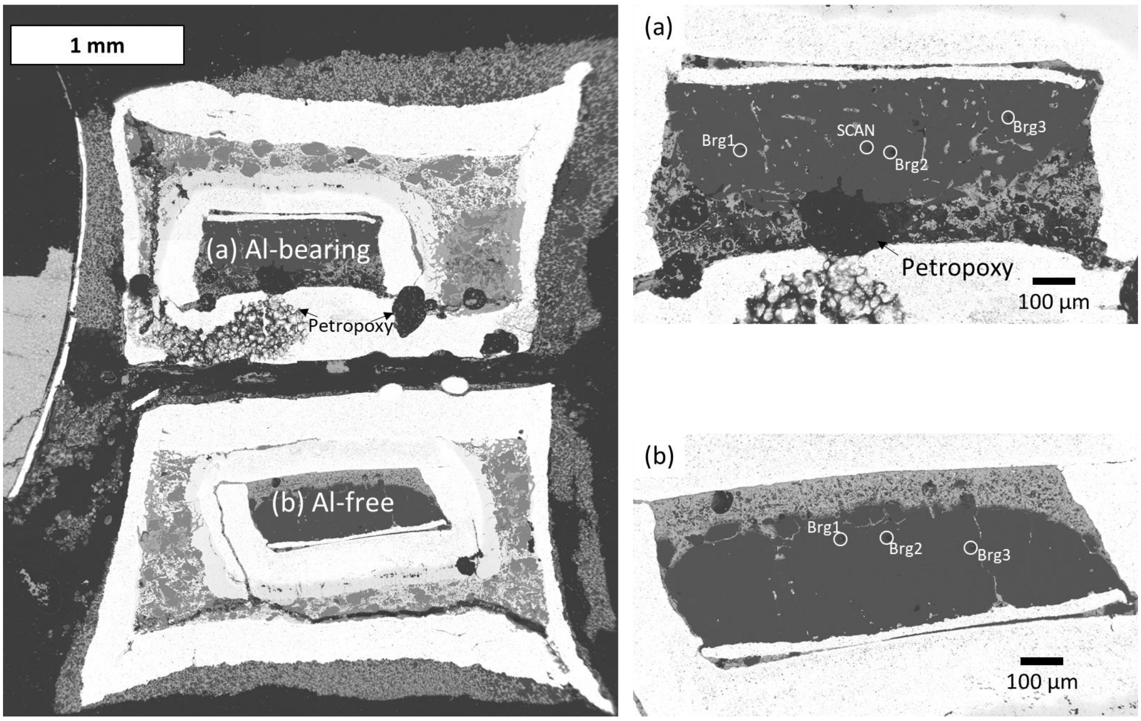

Supplementary Figure 6. BSE images of recovered sample from 28 GPa, 1700 °C (OS3083). The left figure is a whole BSE image of the recovered sample. The right figures are the BSE images of the two samples obtained by FE-SEM after high-resolution SIMS analysis; (a) Al-free system and (b) Al-bearing system. Circles correspond to analysis points. Brg, bridgmanite.

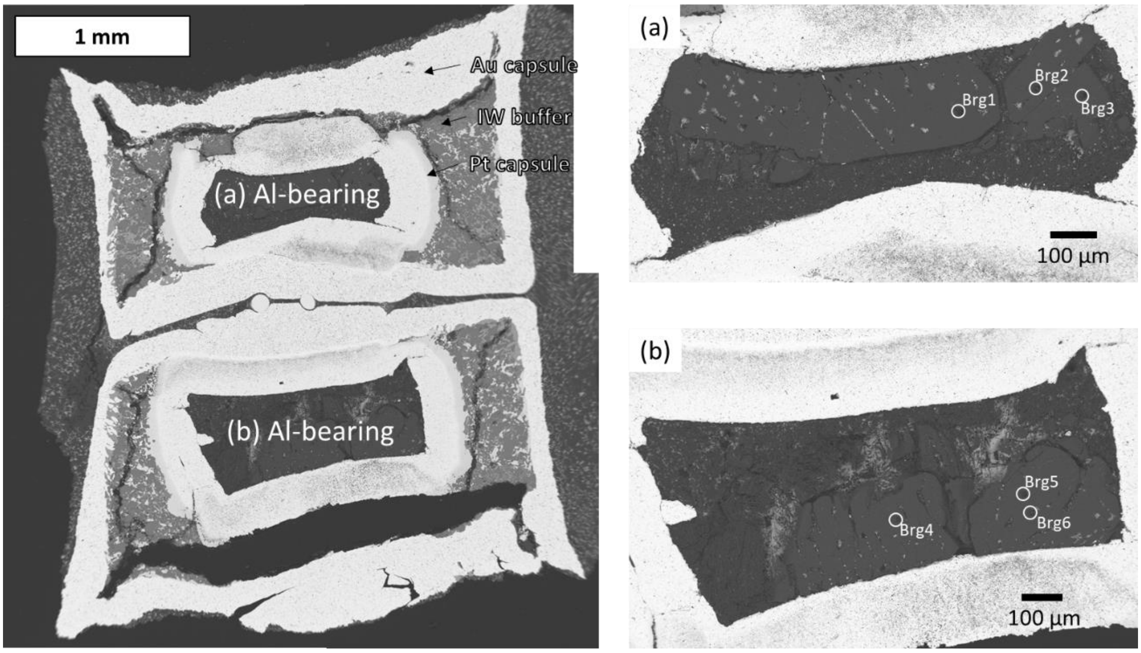

Supplementary Figure 7. BSE images of recovered sample from 28 GPa, 1500 °C (OT2474). The left figure is a whole BSE image of the recovered sample. The right figures are the BSE images of the two samples obtained by FE-SEM after high-resolution SIMS analysis; both (a) and (b) are Al-bearing system. Circles correspond to analysis points. Brg, bridgmanite.

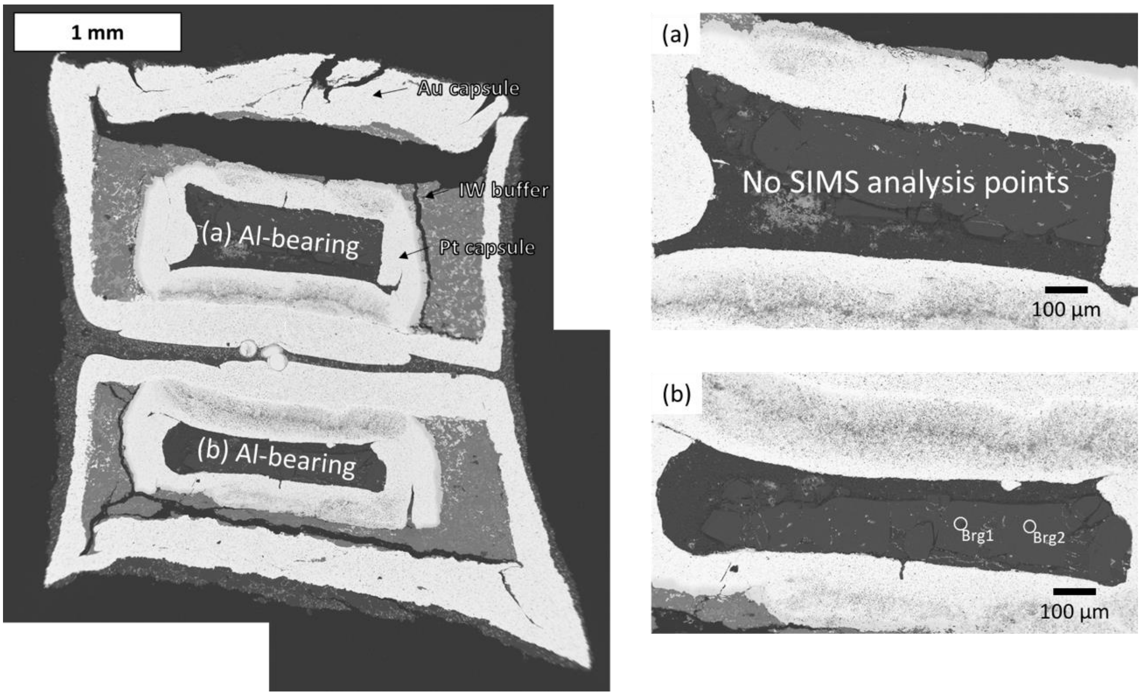

Supplementary Figure 8. BSE images of recovered sample from 28 GPa, 1400 °C (OT2515). The left figure is a whole BSE image of the recovered sample. The right figures are the BSE images of the two samples obtained by FE-SEM after high-resolution SIMS analysis; both (a) and (b) are Al-bearing system. Circles correspond to analysis points. Brg, bridgmanite.

Supplementary Table 1. Chemical compositions of starting materials.

Chemical composition	SiO_2 (wt.%)	Al_2O_3 (wt.%)	MgO (wt.%)	$Mg(OH)_2$ (wt.%)	Fe_2SiO_4 (wt.%)	total
$MgSiO_3$	59.85	0	40.15	0	0	100.00
$MgSi_{0.9}Al_{0.1}H_{0.1}O_3$	53.87	5.08	38.15	2.90	0	100.00

Supplementary Table 2. Experimental conditions and run products in the recovered samples.

Run.no	Starting materials*	Pressure (GPa)	Temperature (°C)	Run duration (min)	Run products
OT2259	a, b	28	1400	120	Brg, St, L
OT2258	a, b	28	1500	120	Brg, St, L
OT2293	a, b	28	1620	120	Brg, St, L
OS3083	a, b	28	1700	120	Brg, St, L
OT2474	b	28	1500	120	Brg, St, L
OT2515	b	28	1400	120	Brg, St, L, Hydrous phase***

Brg: bridgmanite, St: stishovite, Per: periclase, L: liquid (hydrous melt, now quenched crystals).

*Detailed chemical compositions of starting materials are listed in Supplementary Table 1. A: $MgSiO_3$, b: $MgSi_{0.9}Al_{0.1}H_{0.1}O_3$.

** The vitrified bridgmanite was vitrified after the high *P*-*T* experiments.

***Minor unidentified Al-bearing hydrous phases co-existed with bridgmanite.

Supplementary Table 3. Chemical composition of the run products and nitrogen concentrations of bridgmanite (OT2259).

(a) Al-free system

	MgO	SiO_2	FeO	total (wt.%)	^{14}N (ppm)	^{15}N (ppm)
Brg1	39.6	60.4	b. d.	100	13.1	1.7(4)
Brg2	39.3	60.7	b. d.	100	26.6	1.6(4)
Brg3	39.5	60.5	b. d.	100	32.4	1.8(4)

(b) Al-bearing system.

	MgO	Al_2O_3	SiO_2	FeO	total (wt.%)	^{14}N (ppm)	^{15}N (ppm)
Brg1	35.9	2.8	58.8	2.5	100	1.1	2.8(1)
Brg2	38.1	1.3	60.6	b. d.	100	17.9	1.6(4)**

*b. d.: Below detection limit of SEM-EDS.

**Nitrogen solubility was determined by first 7 cycles in high-resolution SIMS (1280 HR2)

Supplementary Table 4. Chemical composition of the run products and nitrogen concentrations of bridgmanite (OT2258).

(a) Al-free system

	MgO	SiO_2	FeO	total (wt.%)	^{14}N (ppm)	^{15}N (ppm)
Brg1	38.9	61.1	b. d. *	100	0.2	2.5(4)
Brg2	38.9	61.1	b. d.	100	0.0	2.8(4)

*b. d.: Below detection limit of SEM-EDS.

Supplementary Table 5. Chemical composition of the run products and nitrogen concentrations of bridgmanite (OT2293).

(a) Al-free system

	MgO	SiO_2	FeO	total (wt.%)	^{14}N (ppm)	^{15}N (ppm)
Brg1	38.9	61.1	b. d.	100	0.0	2.2(4)**
Brg2	38.8	61.2	b. d.	100	0.0	2.8(4)
Brg3	37.5	62.5	b. d.	100	2.1	4.9(4)

*b. d.: Below detection limit of SEM-EDS.

**Nitrogen solubility was determined by the first 14 cycles in high-resolution SIMS (1280 HR2).

Supplementary Table 6. Chemical composition of the run products and nitrogen concentrations of bridgmanite (OS3083).

(a) Al-free system

	MgO	SiO_2	FeO	total (wt.%)	^{14}N (ppm)	^{15}N (ppm)
Brg1	38.8	61.2	b. d.	100	7.4	4.2(5)
Brg2	39.2	60.8	b. d.	100	0	5.7(8)
Brg3	38.1	61.9	b. d.	100	0	3.8(4)

(b) Al-bearing system

	MgO	Al_2O_3	SiO_2	FeO	total (wt.%)	^{14}N (ppm)	^{15}N (ppm)
Brg1	37.3	4.8	58.0	b. d.	100	6.1	5.3(4)
Brg2	38.3	1.7	60.0	b. d.	100	9.0	4.9(5)
Brg3	37.9	2.0	60.1	b. d.	100	22.9	8.0(7)

*b. d.: Below detection limit of SEM-EDS.

Supplementary Table 7. Chemical composition of the run products and nitrogen concentrations of bridgmanite in Al-bearing systems (OT2474).

	MgO	Al_2O_3	SiO_2	FeO	total (wt.%)	^{14}N (ppm)	^{15}N (ppm)
Brg1	Obtained signals were unstable						
Brg2	37.7	4.4	58.0	b. d.	100	18.9	3.4(4)
Brg3	38.1	3.9	58.0	b. d.	100	42.8	4.3(4)
Brg4	38.0	2.9	59.1	b. d.	100	92.6	4.1(4)
Brg5	37.5	3.4	59.1	b. d.	100	13.9	11.9(12)*
Brg6	38.2	2.7	59.1	b. d.	100	16.9	3.9(4)

* Nitrogen solubility was determined by removing first 5 cycles that were unstable signals in high-resolution SIMS (1280 HR2).

**b. d.: Below detection limit of SEM-EDS.

Supplementary Table 8. Chemical composition of the run products and nitrogen concentrations of bridgmanite in Al-bearing systems (OT2515).

	MgO	Al_2O_3	SiO_2	FeO	total (wt.%)	^{14}N (ppm)	^{15}N (ppm)
Brg1	38.8	1.9	59.3	b. d.	100	35.9	6.8(5)
Brg2	39.1	2.3	58.6	b. d.	100	20.7	3.3(4)

*b. d.: Below detection limit of SEM-EDS.
